# Habitat suitability and connectivity implications for the conservation of the Persian leopard along the Iran–Iraq border

**DOI:** 10.1002/ece3.8069

**Published:** 2021-08-30

**Authors:** Shahram Kaboodvandpour, Kamran Almasieh, Navid Zamani

**Affiliations:** ^1^ Department of Environmental Sciences Faculty of Natural Resources University of Kurdistan Sanandaj Iran; ^2^ Department of Zrebar Lake Environmental Research Kurdistan Studies Institute University of Kurdistan Sanandaj Iran; ^3^ Department of Nature Engineering Agricultural Sciences and Natural Resources University of Khuzestan Mollasani Iran; ^4^ Zhooaan Agreen Ecotourism Academy Sanandaj Iran

**Keywords:** core habitats, corridors, habitat fragmentation, large carnivores, Zagros Mountains

## Abstract

Habitat fragmentation has major negative impacts on wildlife populations, and the connectivity could reduce these negative impacts. This study was conducted to assess habitat suitability and structural connectivity of the Persian leopard along the Iran–Iraq border (i.e., the Zagros Mountains) and compare the situation of identified core habitats and connectivity with existing conservation areas (CAs). An ensemble modeling approach resulting from five models was used to predict habitat suitability. To identify core habitats and corridors along the Iran–Iraq border, factorial least‐cost path analyses were applied. The results revealed that topographic roughness, distance to CAs, annual precipitation, vegetation/cropland density, and distance to rivers were the most influential variables for predicting the occurrence of the Persian leopard in the study area. By an estimated dispersal distance of 82 km (suggested by previous studies), three core habitats were identified (two cores in Iran and one core in Iraq). The largest cores were located in the south and the center of the study area, which had the highest connectivity priorities. The connectivity from these cores was maintained to the core within the Iraqi side. Only about one‐fifth of detected core habitats and relative corridors were protected by CAs in the study area. Detected core habitats and connectivity areas in this study could be an appropriate road map to accomplish the CAs network along the Iran–Iraq border regarding Persian leopard conservation. Establishing transboundary CAs, particularly in the core habitat located in the center of the study area, is strongly recommended to conserve existing large carnivores, including the Persian leopard.

## Graphical abstract

Three core habitats of the Persian leopard were detected in the study area (two in Iranian section and one in Iraqi section) and the connectivity from the Iranian cores was maintained to the core within the Iraqi side.

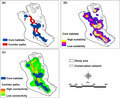

## 1 INTRODUCTION

The pressure of the human population on wildlife habitats results in habitat loss and fragmentation (Almasieh et al., 2019; Bennett, 2003; Berger et al., 2008). Habitat fragmentation is a process in which a large natural habitat is converted into several smaller and spatially separated habitat patches (Bennett, 2003; Ewers & Didham, 2006). This process has significant adverse effects on wildlife populations (Almasieh et al., 2019; Morrison & Mathewson, 2015). The feasible solution for this issue is connectivity, which maintains or facilitates the movement of individuals among habitat patches. The connectivity could lead to gene flow among patches and persistence of species surviving against the occurrence of mass extinction (i.e., sixth mass extinction) (Beier et al., 2007; McRae & Beier, 2007; Soulé, 1986). Least‐cost path modeling (Adriaensen et al., 2003), circuit theory (McRae et al., 2008), centrality analyses (Estrada & Bodin, 2008), factorial least‐cost path density (Cushman et al., 2009), and resistant kernel (Compton et al., 2007) methods have been frequently used to assess landscape connectivity. Furthermore, combining the factorial least‐cost path and cumulative resistant kernel approaches has been used to design core areas and corridors for large carnivores (Cushman et al., 2013; Khosravi et al., 2018; Shahnaseri et al., 2019).

Large carnivores are sensitive to habitat fragmentation due to their vast home range and often low population density (Calvignac et al., 2009; Mohammadi et al., 2021; Noss et al., 1996). Therefore, they could be considered the focal species in the landscape (Almasieh et al., 2016; Beier et al., 2008). Large carnivores are top predators and play an important role as keystone species on top of the natural food chains (Crooks, 2002). Therefore, reduction in their population size and extinction of these species will dramatically lead to change in ecosystems’ structural and ecological processes and their dependent biotic communities (Crooks, 2002; Estes et al., 2011). By conserving the large carnivores as umbrella species, other species (i.e., small carnivores, mammals, other vertebrates, invertebrates, and plants) will be covered and protected (Beier et al., 2008; Sampson, 2013).

The leopard (*Panthera pardus* Linnaeus, 1758) is widely distributed in the continents of Asia and Africa and is known as the most widespread distribution among felids (Family: Felidae) (Gavashelishvili & Lukarevskiy, 2008; Nowell & Jackson, 1996). However, its population has been reduced and is being isolated because of human population pressure and the impact of their related activities (Thorn et al., 2013), habitat fragmentation (Selvan et al., 2014), illegal hunting (Datta et al., 2008), and prey decline (Hatton et al., 2001). By decreasing population size, the leopard has been categorized as a vulnerable (VU) species according to the IUCN Red List (Stein et al., 2020). Nine subspecies have been identified for the leopard so far (Uphyrkina et al., 2001), one of them is the Persian leopard (*P*. *pardus saxicolor* Pocock, 1927), distributed in southwestern Asia (Appendix S1: Figure S1). The most significant distribution and population of the Persian leopard have been reported in Iran (Kiabi et al., 2002). Although the Persian leopard has been categorized as an endangered (EN) species in the IUCN Red List (Khorozyan, 2016), more recently the assessment does not appear any longer on the IUCN Red List due to data contradictory and lack of up‐to‐date information (Stein et al., 2020). The Zagros Mountains forming from the mountain ranges in Iran to the northeast of Iraq (Kurdistan region) has been reported as one of the cross‐border habitats of the Persian leopard (Avgan et al., 2016; Breitenmoser et al., 2010), but our knowledge about the Persian leopard habitat suitability and its existing core habitats in the region is so little.

Habitat suitability models (HSMs) (Guisan & Zimmermann, 2000) were frequently used by wildlife researchers to predict core habitats and corridors, especially for the cryptic nocturnal animals such as large carnivores (Beier et al., 2007). Several studies have been done on the habitat modeling of the Persian leopard in Iran (e.g., Ahmadi et al., 2020; Ashrafzadeh et al., 2018, 2020; Farhadinia et al., 2015; Hosseini et al., 2019; Khosravi et al., 2018, 2019, 2021). However, none of them have focused on the west of Iran with sufficient occurrence points, particularly near borders. Thus, the west of Iran could be assumed to be the unknown habitat of the Persian leopard in Iran. In addition to the brown bear (*Ursus arctos*), the Persian leopard is the top predator along the mountainous areas of Iran–Iraq border (Almasieh et al., 2019; Karami et al., 2015), which could be identified as focal species in the mentioned region. The Persian leopard is a mobile species and therefore, its survival chance is highly dependent on the rate of conservation management in its natural habitats, particularly along the political borders (Hosseini et al., 2019). In addition, the conservation of the Persian leopard depends on the accurate detection of the core habitats and their connectivity (Mohammadi et al., 2021). Due to special conditions of political borders, with proper use of HSMs and even by the limited extensive field surveys, the prediction of core habitats and corridors of the Persian leopard could be conducted. This study focused on (a) habitat suitability of the Persian leopard along the Iran–Iraq border, (b) detecting possible connectivity between core habitats, (c) determination of connectivity priority of core habitats, and (d) the comparison of identified core habitats and their connectivity with existing conservation areas (CAs) along the Iran–Iraq border.

## 2 MATERIALS AND METHODS

### 2.1 Study area

**FIGURE 1:**
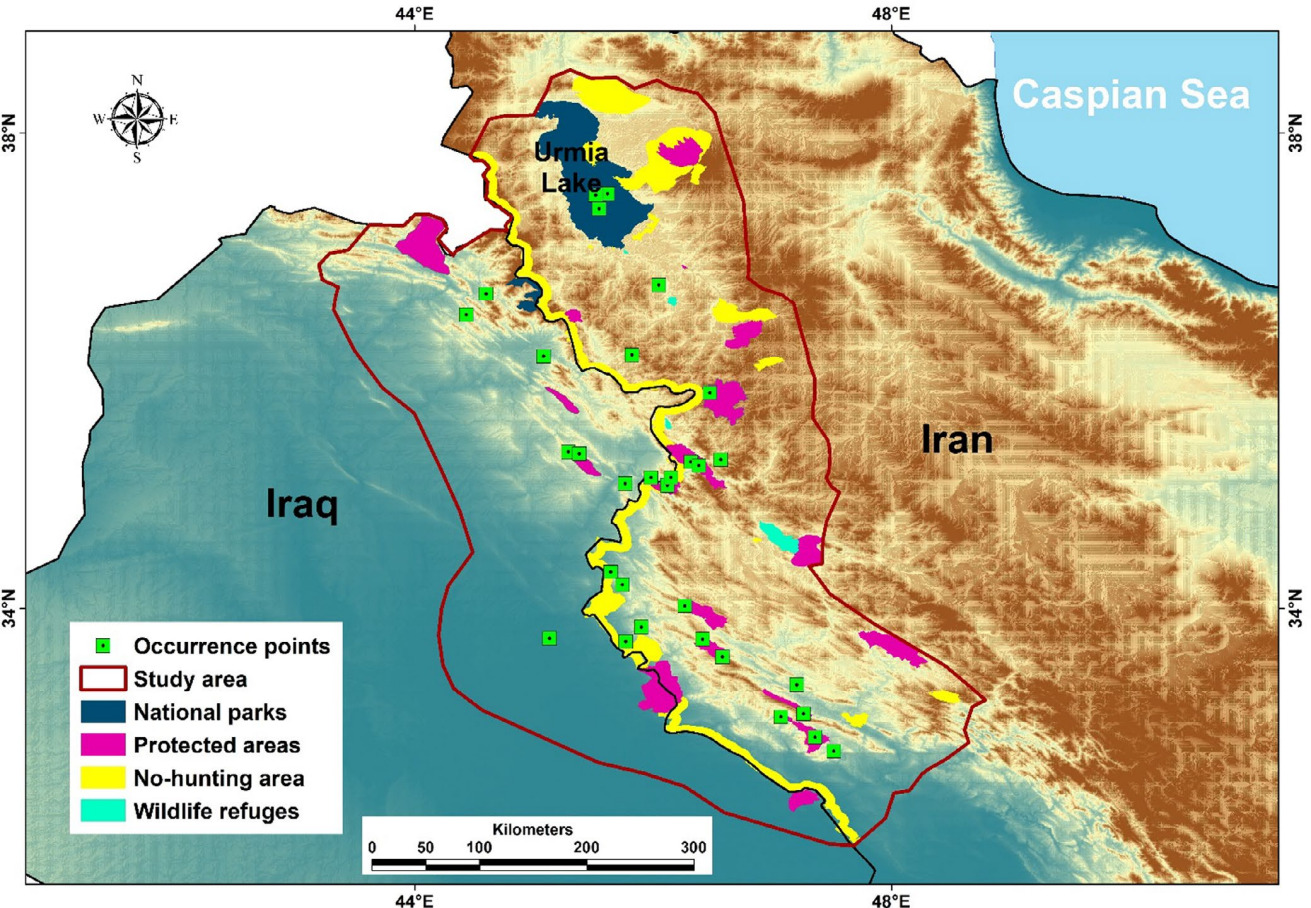
Study area along the Iran–Iraq border, conservation areas, and occurrence points of the Persian leopard

There is a 1,450‐km common border between Iran and Iraq. The southern part (approximately 300 km) includes arid plains entirely with no records of the occurrence of the Persian leopard. Therefore, this part was excluded and the remaining 1,050 km was considered. Moreover, due to the Persian leopard's presence in northwestern Iran and the probability of habitat connectivity with the Iraqi section, an additional 100‐km border up to Urmia Lake was considered as well. Based on six tracked Persian leopards in the northeast of Iran with a maximum cruising radius of 81.6 km (Farhadinia et al., 2018), a 100 km buffer zone was selected around each occurrence point of the Persian leopard, and a minimum convex polygon formed the study area (Figure 1). The study area, an area of approximately 195,000 km^2^ situated in the central and northern Zagros Mountains, is a part of the Irano‐Anatolian global biodiversity hotspot (Mittermeier et al., 2005). CAs in the study area include two National Parks (NPs), four Wildlife Refuges (WRs), 19 Protected Areas (PAs), and 11 No‐Hunting Areas (NHAs). In addition, the width of seven kilometers on the border of the Iranian side is considered NHA (Appendix S1: Figure S2 and Table S1). NPs have the highest conservation rank, and WRs and NPs have the second and third ranks, respectively. NHAs have the lowest conservation rank and were established to control animals’ poaching (Ahmadi et al., 2020). CAs, including NPs, WRs, Pas, and NHAs, are close to the II, III, IV, and IV–VI of the IUCN categories, respectively (Ahmadi et al., 2020; Lausche & Burhenne‐Guilmin, 2011).

### 2.2 Data collection

Occurrence points of the Persian leopard were collected from three different sources: (1) the reports of the guards and external officers of the Department of Environment (DoE) and previous studies during 2000–2020, (2) random field surveys on the northern part of the study area with a high probability of the species occurrence during 2017–2020 and recording the occurrence points by using the Global Positioning System with error <10 and (3) study by Avgan et al. (2016) with occurrence points from 2008 to 2014. Seventy‐four (74) occurrence points of the Persian leopard were recorded in the sources as mentioned earlier (16, 50, and 8, respectively). To minimize spatial autocorrelation, a radius of 5 km (according to the mean maximum distance moved by the Persian leopard per day, i.e., 5 km [Ahmadi et al., 2020; Farhadinia et al., 2015; Ghoddousi et al., 2010]) was considered around each occurrence point to spatial filtering by using the Spatially Rarify Occurrence Data tool in the SDMtoolbox (Brown, 2014). Finally, 31 occurrence points have remained for habitat modeling of the Persian leopard in the study area (Appendix S1: Table S2).

### 2.3 Environmental variables

According to previous studies, environmental variables, including topographic and climatic variables, land cover, water resources, prey availability, and anthropogenic variables, were used for habitat modeling of the Persian leopard (Appendix S1: Table S3). Digital Elevation Model (DEM) from the 30‐m Shuttle Radar Topography Mission (SRTM, downloaded from http://earthexplorer.usgs.gov) was used to calculate the topographic roughness index (variance of elevation value of DEM᾽s cells in the 5‐km neighborhood) by utilizing Geomorphometry and Gradient Metrics Extension (Evans et al., 2014) in ArcGIS version 10.3. Two climatic variables, frequently used by previous studies (Appendix S1: Table S3), selected to predict the distribution of the Persian leopard are annual mean temperature (BIO1) and annual precipitation (BIO12), (http://worldclim.org; Fick & Hijmans, 2017).

A global land‐cover map (GlobCover version 2.3) with 23 cover types was used for habitat modeling (ESA, 2009). Two out of 14 cover types in the study area, with the highest probability of the species occurrence, were chosen for habitat modeling: (1) mosaic vegetation (grasslands, shrublands, and forests) (20%–50%)/cropland (50%–70%) and (2) mosaic grassland (50%–70%)/forest‐shrubland (20%–50%). In addition, these two cover types were used by previous research for habitat modeling of the Persian leopard in the transboundary area (i.e., Farhadinia et al., 2015). A circle‐moving window with a 5‐km radius was used to create density maps of these two cover types. The 16‐day composite MODIS data (MOD13A1 V6 map at 500‐m cell size; http://earthexplorer.usgs.gov) was used to calculate the Normalized Difference Vegetation Index (NDVI) according to the mean values of the year 2019. Given the importance of water resources for animals (Almasieh et al., 2019), distance to rivers (DoE, 2018; OCHA, 2018) was also included in habitat modeling of the Persian leopard by using Euclidean Distance tool in ArcGIS. Prey availability should be considered in habitat modeling of the Persian leopard as a biotic factor for the power of prediction (Khosravi et al., 2021). As CAs support the three main prey species of the Persian leopard in the study area, including bezoar goat (*Capra aegagrus*), mouflon (*Ovis gmelini*), and roe deer (*Capreolus capreolus*), distance to CAs was created as a surrogate of prey availability. Due to the acute adverse effects of roads on felids (Mohammadi et al., 2018), distance to roads (DoE, 2018; OCHA, 2018) and also distance to villages (DoE, 2018; OCHA, 2018) as anthropogenic variables were used for habitat modeling of the Persian leopard in the study area.

To check multicollinearity among variables, pairwise Pearson correlation was used to exclude variables with correlation coefficient of >70%. Ten variables were chosen for the Persian leopard habitat modeling with a lower correlation coefficient of 70%. The second method to reduce multicollinearity among variables was the Variance Inflation Factor (VIF) of the dataset, which was checked by using the usdm package (Naimi et al., 2014) in the R (R Core Team, 2019) to exclude variables (selected in the previous step) with VIF >3 (threshold suggested by Zuur et al., 2010). VIF for the 10 variables was lower than three (Appendix S1: Table S4). Therefore, all of these variables were included in the habitat modeling.

### 2.4 Habitat modeling

An ensemble modeling approach was used to predict habitat suitability of the Persian leopard by using the R package biomod2 (Thuiller et al., 2016). The ensemble model increases the model's accuracy by combining predictions of different models and fitting several suitability models rather than a single model with an uncertain prognosis (Araújo & New, 2007; Shahnaseri et al., 2019). This approach has also been considered in other studies related to the habitat suitability of the Persian leopard (Ahmadi et al., 2020; Ashrafzadeh et al., 2018; Farhadinia et al., 2015; Khosravi et al., 2018). Ten prediction models were implemented, including four regression‐based models (Generalized Linear Model [GLM], Generalized Additive Model [GAM], Multivariate Adaptive Regression Splines [MARS] and Flexible Discriminant Analysis [FDA]), five machine‐learning models (Random Forest [RF], Maximum Entropy [MaxEnt], Generalized Boosting Model [GBM], Classification Tree Analysis [CTA], and Artificial Neural Network [ANN]), and one profile model (Surface Range Envelop [SRE]). The primary analysis for habitat modeling was carried out by using 10 models, then, five models (i.e., GLM, MARS, RF, MaxEnt, and GBM) with AUC and True Statistic Skill (TSS) thresholds higher than 0.9 and 0.75 (Eskildsen et al., 2013) were chosen (Appendix S1: Table S5). Barbet‐Massin et al. (2012) suggested that the best occurrence/pseudo‐absence ratio was one‐tenth for most of the models in biomod2 by applying 20 replicates for each model and equal weights of occurrence and pseudo‐absence points. Regarding the number of occurrence points (i.e., 31), 300 pseudo‐absence points were randomly created across the whole study area and outside of the 5‐km radius circle (radius of a circular home range of about 80 km^2^) around each occurrence point. The analyses were carried out by applying 20 replicates for each model to obtain conservative results. In addition, a prevalence of 0.5 (means the exact weights of occurrence and pseudo‐absence points) was considered (Calambás‐Trochez et al., 2021). The variables’ contribution for each model was calculated in Biomod2. In addition, response curves of occurrence points to the variables for each model were determined in the study area.

The ensemble suitability map was converted into a resistance map according to the method of Wan et al. (2019). First, the ensemble map was rescaled to a 0–1 map using the linear method in Rescale by Function tool in ArcGIS. Then, a negative exponential function (*R* = 1,000^(−1×HS)^, Mateo‐Sánchez et al., 2015) was used to create the resistance map, where R represents the cost resistance value assigned to each pixel and HS represents the predicted habitat suitability derived from the ensemble suitability model described above (Wan et al., 2019). Finally, the resistance values were rescaled to a range between 1 and 10 by linear interpolation; when HS was 1, the minimum resistance became 1, and when HS was 0, the maximum resistance became 10 (Wan et al., 2019).

### 2.5 Connectivity modeling

Structural connectivity (hereafter, connectivity) modeling was carried out by using Universal Corridor (UNICOR) software (Landguth et al., 2012) and two sets of connectivity predictions consisting of (1) resistant kernels and (2) factorial least‐cost paths. Resistant kernels are an algorithm that calculates the resistance cost‐weighted dispersal around each source point up to a dispersal threshold defined by the user (Compton et al., 2007; Mohammadi et al., 2021). An incidence function of the rate of organism movement through every pixel in the landscape was provided as a function of the density and number of source points, the dispersal ability of the species, and the resistance of the landscape (Cushman et al., 2013). The second set of connectivity prediction was implanted in the UNICOR simulator applying Dijkstra's algorithm to resolve the single‐source shortest path issue from every mapped species occurrence point on a landscape to every other occurrence points (Landguth et al., 2012). The analysis produces predicted least‐cost path routes from each source point to each destination point.

According to the study of Farhadinia et al. (2018), the distance threshold of 82,000 was used in resistant kernel analyses, which represents movement ability of 82 km. The buffered least‐cost paths were then combined through summation to produce the connectivity map between all pairs of occurrence points (Cushman et al., 2009). The connectivity map was used to identify core habitats of the Persian leopard with the selected scenario. The contiguous map of patches with resistant kernel values was converted to a categorical map based on >10% of the highest records for the species (Ahmadi et al., 2020; Ashrafzadeh et al., 2020; Cushman et al., 2013). Furthermore, this was carried out for factorial least‐cost paths. The coverage of CAs with core habitats and corridors of the Persian leopard was calculated separately in the study area.

### 2.6 Connectivity prioritization of core habitats

The probability of connectivity (dPC; Saura & Pascual‐Hortal, 2007) was measured to create habitat patches prioritization based on dPC including dPCintra, dPCflux, and dPCconnector (Saura & Pascual‐Hortal, 2007) by using Conefor 2.6 software (Saura & Torné, 2009). dPCintra only measures intrapatch connectivity and does not depend on species dispersal distance, while dPCflux measures dispersal flux and depends on the patch area and its position within the landscape. dPCconnector depends only on the topological position of a patch within the landscape (Saura & Rubio, 2010), which quantifies the importance of the node as a stepping stone for dispersal and facilitates the dispersal between excessively far nodes (Avon & Bergès, 2016; Saura & Rubio, 2010). To prepare the data for Conefor, the 10% of the highest resistance kernel recorded for the Persian leopard was used as a threshold to create categorical core patches (Ahmadi et al., 2020). The dispersal distance scenario of 82 km was considered for identified core habitats. Moreover, Conefor Input ArcGIS extension (http://www.jennessent.com/arcgis/conefor_inputs.htm) was applied to prepare Conefor software inputs (node and distance files).

## 3 RESULTS

### 3.1 Habitat suitability

**FIGURE 2:**
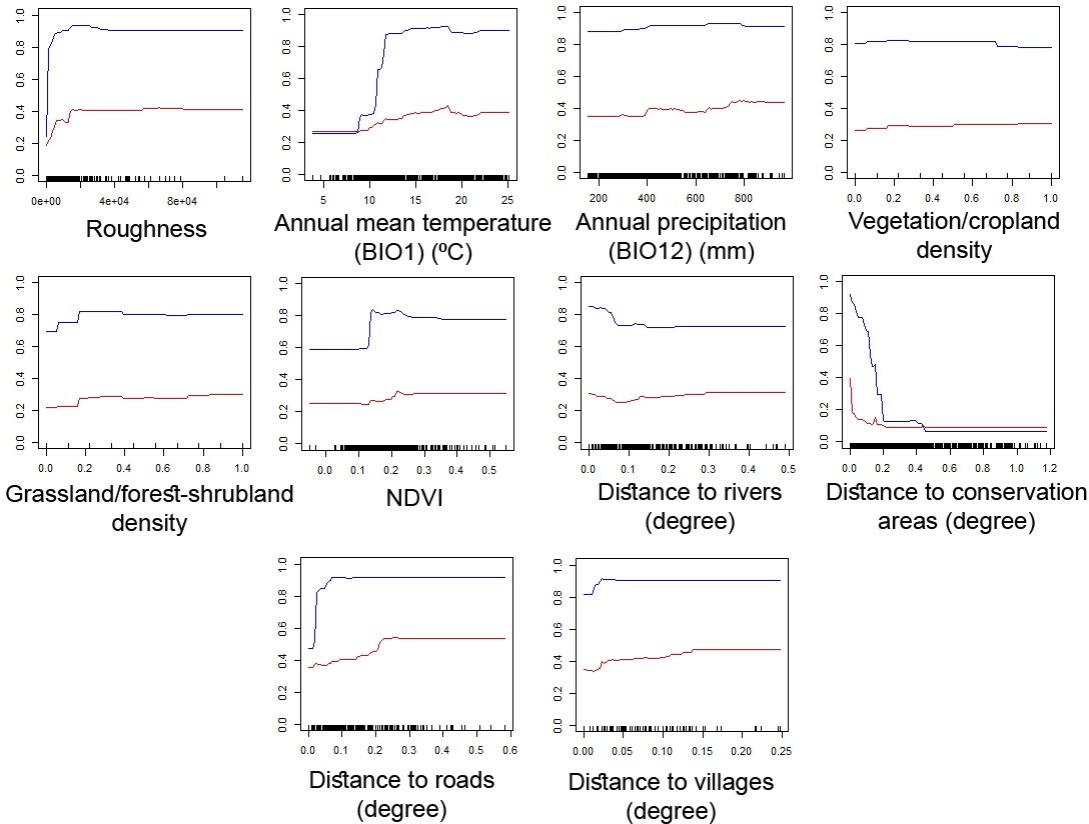
Response curves of occurrence points of the Persian leopard to the environmental variables in the study area (the two most accurate models of RF [red] and GBM [blue] were considered) (*Y*‐axis represents the probability of the Persian leopard occurrence) (each 0.1 degrees in the study area is approximately equal to 14.4 km)

**FIGURE 3:**
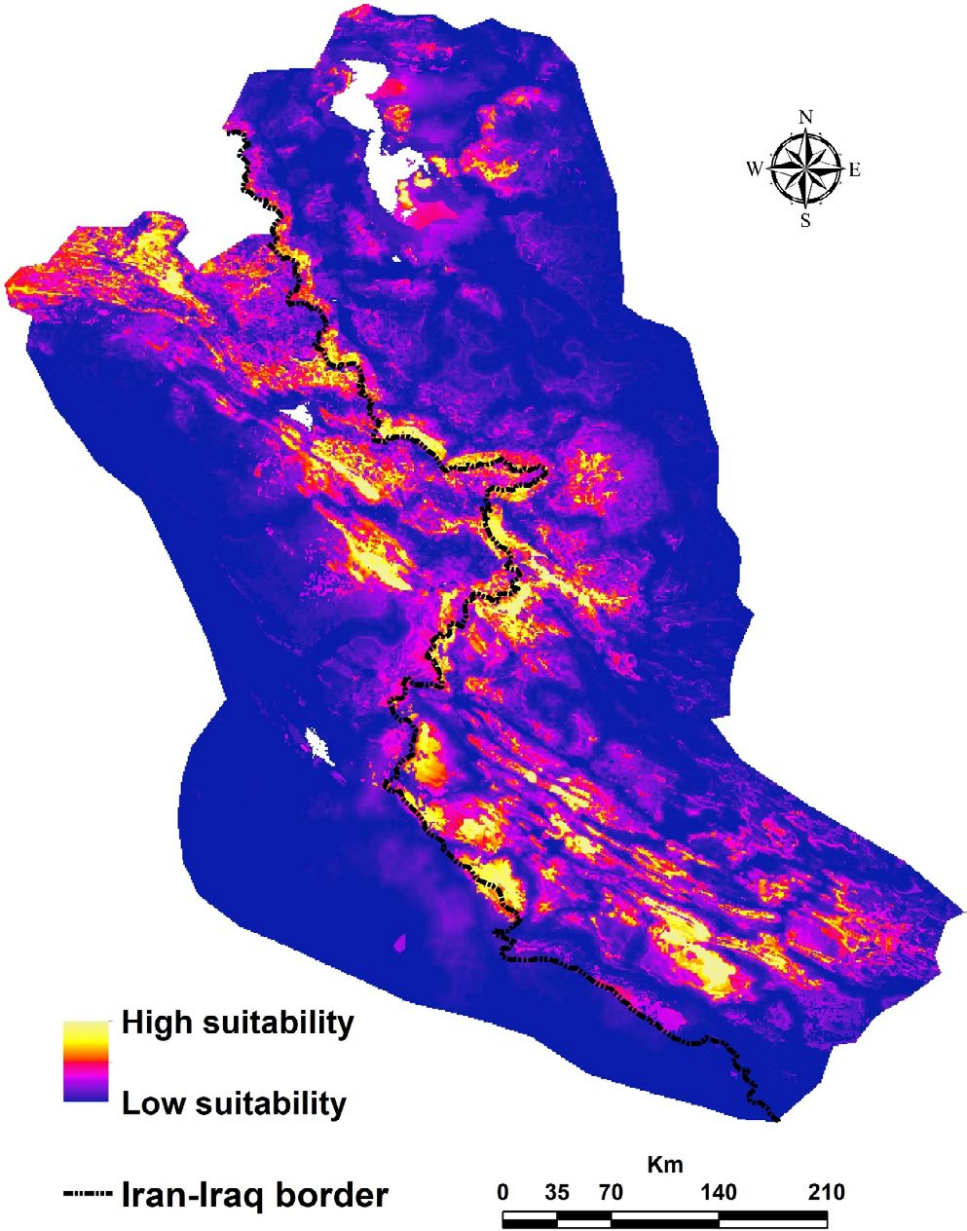
Ensemble habitat suitability map for the Persian leopard in the study area based on the five optimal models of GLM, MARS, RF, MaxEnt, and GBM

Based on the five obtained optimal models, variables of topographic roughness, distance to CAs, annual precipitation, vegetation/cropland density, and distance to rivers were the most influential variables for predicting the occurrence of the Persian leopard (Appendix S1: Table S6). By increasing topographic roughness, the probability of the occurrence of the Persian leopard increased. The optimal range of mean temperature and annual precipitation for the occurrence of the Persian leopard were 10–25°C and 400–800 mm, respectively. As NDVI in both natural (i.e., grasslands, forests, and shrublands) and agricultural vegetation (croplands) increased, the likelihood of the occurrence of the Persian leopard increased. As distance to rivers and distance to CAs increased, the likelihood of the occurrence of the Persian leopard decreased. Finally, by increasing distance to roads and distance to villages, the probability of the occurrence of the Persian leopard increased gradually and then stabilized (Figure 2). Ensemble suitability map showed that monotonous areas in the southwest, center, and northeast of the study area had the highest suitability for the Persian leopard (Figure 3). All HSMs had a similar pattern with higher suitable areas (GLM) versus lower suitable areas (RF and GBM) (Appendix S1: Figure S3).

### 3.2 Core habitats and corridors

**FIGURE 4:**
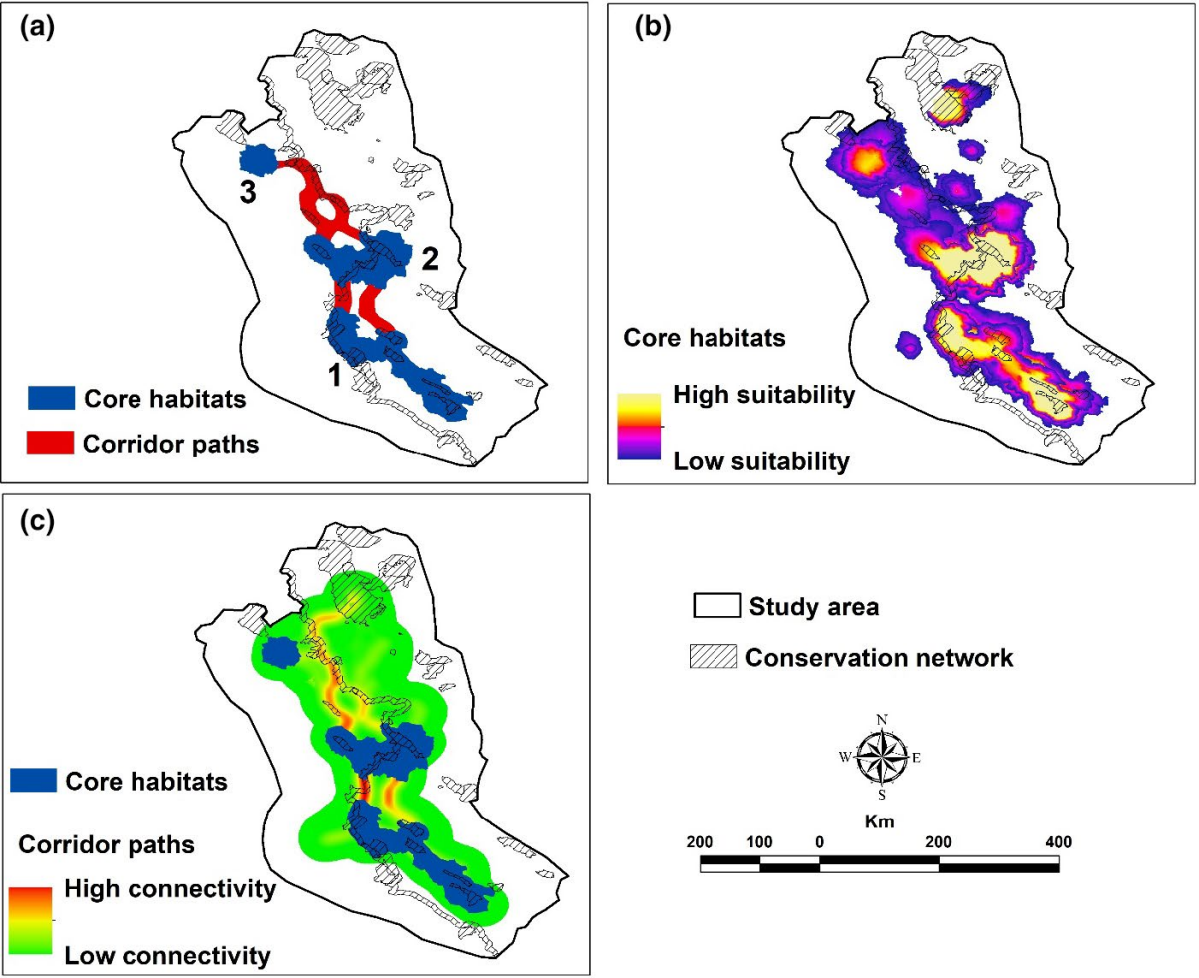
Core habitat and corridor maps for the Persian leopard in the study area (a: categorical core habitats and corridor paths, b: contiguous core habitats, and c: contiguous corridor paths)

The most important core habitats were located in the south and central parts of the study area (Figure 4). In the estimated dispersal distance of 82 km, three core habitats were identified (Appendix S1: Table S7). The largest habitat patch was Core1, located in the southern part of the study area (about 11,500 km^2^). The second‐largest habitat patch was Core2, located in the center of the study area (about 9,300 km^2^) (Figure 4, Appendix S1: Table S7). These two important large patches covered almost 11% of the study area. Seven PAs, three NHAs, and the width of seven kilometers on the border of the Iranian side were located in Core1 and Core2 (Appendix S1: Table S7). Only 19.47% of the predicted core habitats were covered by CAs (Appendix S1: Table S8).

The connectivity for the Persian leopard in the study area was maintained between core habitats from south to north (Figure 4). The highest connectivity was predicted to be between the south and center of the study area between Core1 and Core2. This connectivity had two branches; the one near the Iran–Iraq border had higher connectivity. High connectivity was maintained between Core2 and Core3 with two branches; one within the Iraqi section with higher connectivity and another along the Iran–Iraq border, which connect together along the border before they get to Core3 (Figure 4). Overall, 19.92% of corridors were covered by CAs, but the most predicted corridors were located outside of CAs (Figure 4; Appendix S1: Table S8).

### 3.3 Connectivity prioritization of core habitats

Based on dPC index, Core1 and Core2 had the highest contribution in habitat connectivity (Appendix S1: Figure S4). According to the results of dPCintra, Core1 and Core2 had the highest intrapatch connectivity. Based on the results of dPCflux, both Core1 and Core2 had approximately similar values and had the highest flux according to patch area and the position within the landscape. Core2 had the highest contribution as a stepping stone (Appendix S1: Figure S4).

## 4 DISCUSSION

This study was carried out in the northern part of the Zagros Mountains in adjacent areas of the Iran–Iraq border with the highest diversity of mammals in Iran (Yusefi et al., 2019). By assuming 82 km cruising radius of the Persian leopard (Farhadinia et al., 2018), there are three core habitats available for the species, two of which are located on the Iranian side (Figure 4). The optimal connectivity was detected between cores in the south and center of the study area. These cores had the highest contribution to habitat connectivity. In addition, connectivity was maintained between two cores within Iran and one core in Iraq. This could be due to the reasonably developed CAs network (Appendix S1: Figure S2 and Table S1) and the larger and closer simulated suitable habitats on the Iranian side (Figure 3).

### 4.1 Contribution of variables

The Persian leopard preferred areas with greater roughness and density of vegetation and areas with high prey availability and closer distance to the rivers with moderate to relatively high annual precipitation. Farhadinia et al. (2015) reported that topographic roughness was the most important habitat variable out of the 14 used variables for habitat modeling of the Persian leopard in the Iranian Caucasus. Hosseini et al. (2019) obtained similar results for habitat modeling of the Persian leopard in the northeast of Iran. Furthermore, Khosravi et al. (2019) mentioned that topographic roughness was a key habitat variable for the Persian leopard in the central mountains of Iran since it provides more preys, fewer human footprints and milder temperature. Altogether, topographic roughness and prey availability are the most interrelated factors for surviving the Persian leopard in the study area.

Vegetation, including grassland, shrubland, and forest, is also a key habitat variable for leopards (Hunter, 2013). Rangelands (mainly grasslands) were the second important variable for habitat modeling of the Persian leopard in central Iran (Khosravi et al., 2019). Woodland and forest‐shrubland mosaic were important cover types for the Persian leopard distribution in the studies of Farhadinia et al. (2015) and Hosseini et al. (2019), respectively. Cropland could be an important habitat variable indirectly. The Persian leopard has a moderate tolerance to human presence (Ahmadi et al., 2020; Soofi et al., 2019). They might approach villages and croplands at times to chase livestock and domestic dogs, which results in the human–leopard conflict (Babrgir et al., 2017; Naderi et al., 2018; Parchizadeh & Adibi, 2019). Rivers provide the Persian leopard water and facilitate its predation due to the higher prey diversity and abundance around riversides. Local guards have reported that Persian leopard hunt roe deer near the rivers in Buzin and Marakhil PA. Four recorded Persian leopard presence points were located near rivers of mountainous areas, particularly in the Shaho‐Kosalan PA, during the field survey in this study. Moreover, rivers with higher vegetation density and consequently more cover and food guarantee the Persian leopard and other animals’ chance of survival (Beier et al., 2007).

### 4.2 Core habitats, connectivity, and CAs

Three core habitats were spotted for the predicted dispersal of 82 km of the Persian leopards in the study area. Two cores occur on the Iranian side of the border (Core1 and Core2), while Core3 and a part of Core2 are located on the Iraqi side. Core1 is the most significant core habitat in the study area. Ashrafzadeh et al. (2020) also introduced Core1 in their study. In this study, this core has been extended to the Iran–Iraq border due to the greater number of occurrence points along the Iran–Iraq border. Although Core1 spreads close to the Iran–Iraq border, it cannot embrace connectivity to the Iraqi side due to the relatively vast plain areas next to Core1 on the Iraqi side. The second largest core would be Core2, which crosses the Iran–Iraq border. Core2 was also introduced by Ashrafzadeh et al. (2020) with high similarity. However, Core2 was not limited to Iran and included the Iraqi side near the Iran–Iraq border. Core2 covered three CAs (Buzin and Marakhil PA, and Shaho‐Kosalan PA on the Iranian side, and Qara‐Dagh PA on the Iraqi side). It seems that the Persian leopard's ability to disperse in this core habitat is mainly owed to the permanent monitoring patrol by border guards from both sides. High connectivity was detected on the Iranian side (south and center of the study area between Core1 and Core2). In addition, high connectivity was observed between Core2 and Core3 on the Iraqi side because of mountainous areas located in the northeast of Iraq. The core habitat in the islands of Urmia Lake was not considered because this area is not a natural habitat for the Persian leopard and no population was established. It is unlikely to do such a thing in the future as the lake is currently recovering. Ashrafzadeh et al. (2020) showed that connectivity was maintained from Core2 to the northwest of Iran through the Iran–Iraq border. Our corridors were near the border, but by extending Core2 to the Iraqi side, the connectivity entered Iraq. Our results suggest that limiting the habitat suitability to a political country might not reflect the fact well.

Only about 19.5% of core habitats are covered by CAs within the study area. Ashrafzadeh et al. (2020) reported that 58% of core habitats for the Persian leopard are protected in other parts of Iran. This means that the conservation action plan for this species must be reconsidered in the west of Iran. The Persian leopard is exposed to poaching and reduction of prey outside the CAs (Ahmadi et al., 2020; Ashrafzadeh et al., 2020; Khosravi et al., 2019). More CAs are needed to protect the Persian leopard effectively along the Iran–Iraq border. Furthermore, only about 20% of detected corridor habitats are covered by CAs and the same action is needed. Reconsideration of CAs network (particularly between Core1 and Core2) is needed to ensure gene flow and individual movement between core habitats (Opdam & Wascher, 2004; Ruiz‐González et al., 2014) as well as increasing the survival rate of the Persian leopard in the study area.

### 4.3 Connectivity priority of core habitats

Core1 and Core2 had the highest connectivity priority and the highest intrapatch connectivity between patches. Therefore, Core1 and Core2, and the corridor habitat between them have to be covered by CAs immediately. Core 1 and Core2 are also identified as the core habitats with the highest flux because of their position in the study area, and therefore, they need special attention. Core2 is the only connectivity route between Core1 in the south and Core3 in the northeast of the study area. Therefore, Core 2 could be assumed as a stepping‐stone core in the study area.

### 4.4 The implications for the conservation

Nowadays, human footprints are found everywhere. Most natural habitats are occupied or converted by humans and only small habitat patches remain (Berger et al., 2008). Large carnivores such as the Persian leopard need vast and integrated natural habitats to meet their different biological requirements (Calvignac et al., 2009). Therefore, large patches are necessary to keep the Persian leopard alive and protecting them is an urgent priority (Crooks et al., 2011; Hilty et al., 2006). Detected core habitats and connectivity areas in this study could be an appropriate road map to accomplish the CAs network in the west of Iran regarding the Persian leopard conservation. This has been suggested for other sympatric large carnivores (e.g., the brown bear) in the center of the study area (Core2) (Almasieh et al., 2019). Developing new CAs is strongly recommended on the Iraqi side in Core2 to cover the whole patch as there are two CAs (Buzin and Marakhil PA, and Shaho‐Kosalan PA) on the Iranian side of the Iran–Iraq border. Political collaboration between countries of Iran and Iraq and designing efficient transboundary CAs could protect the core habitats and help the safe connectivity, which leads to the survival of large carnivores along the border of countries. This issue was recommended by the previous studies on the habitat modeling and connectivity of the Persian leopard in the country borders (i.e., Iran‐Armenia‐Azerbaijan by Farhadinia et al. (2015) and Iran‐Turkmenistan‐Afghanistan by Hosseini et al. (2019)).

## 5 CONCLUSION

This study was conducted in the most unknown habitat for the Persian leopard along the Iran–Iraq border. Three core habitats were identified with the highest connectivity priorities in the south and center of the study area. This confirmed that habitat fragmentation occurred and this has to be compensated by detecting and establishing the potential corridor habitats and developing the CAs network. Only about one‐fifth of detected core habitats and relative corridors are covered by CAs in the study area. Reconsideration and development of CAs network in the study area (in both countries) are strongly recommended to fill gaps and guarantee the survival of the Persian leopard in the region.

## CONFLICT OF INTEREST

The authors declare no conflict of interest.

## AUTHOR CONTRIBUTION

**Shahram Kaboodvandpour:** Conceptualization (equal); Methodology (equal); Resources (equal); Supervision (equal); Validation (equal); Writing‐original draft (equal); Writing‐review & editing (equal). **Kamran Almasieh:** Conceptualization (equal); Methodology (equal); Supervision (equal); Validation (equal); Writing‐original draft (equal); Writing‐review & editing (equal). **Navid Zamani:** Conceptualization (equal); Methodology (supporting); Resources (equal); Validation (supporting); Writing‐original draft (equal); Writing‐review & editing (equal).

## Supporting information

Appendix S1

## Data Availability

Data used for the analysis are uploaded in a Dryad repository (https://doi.org/10.5061/dryad.c2fqz618p).
