# Supplementary material for: Habitat suitability and connectivity implications for the conservation of the Persian leopard along the Iran–Iraq border
Source: Ecol Evol. 2021 Aug 30;11(19):13464–74. doi: 10.1002/ece3.8069 (PMC8495822; doi:10.1002/ece3.8069)
Supplement: Supplementary file 1 — Appendix S1 [file ECE3-11-13464-s001.docx]

**APPENDIX**

**FIGURES**


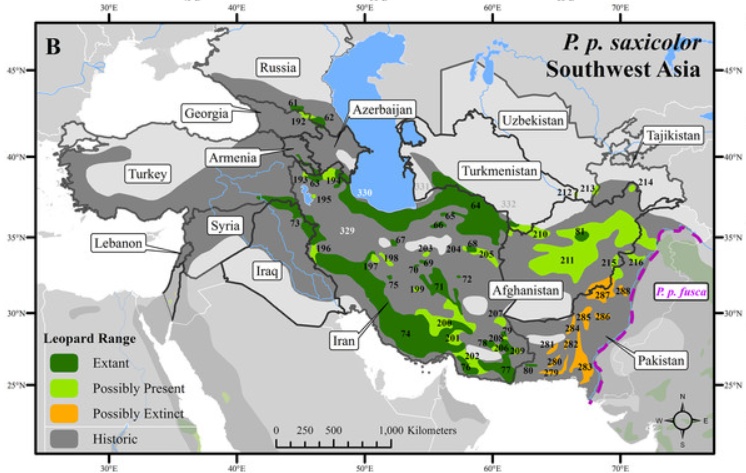


FIGURE S1 Global distribution of the Persian leopard (*Panthera pardus saxicolor*) (After, Jacobson et al., 2016)


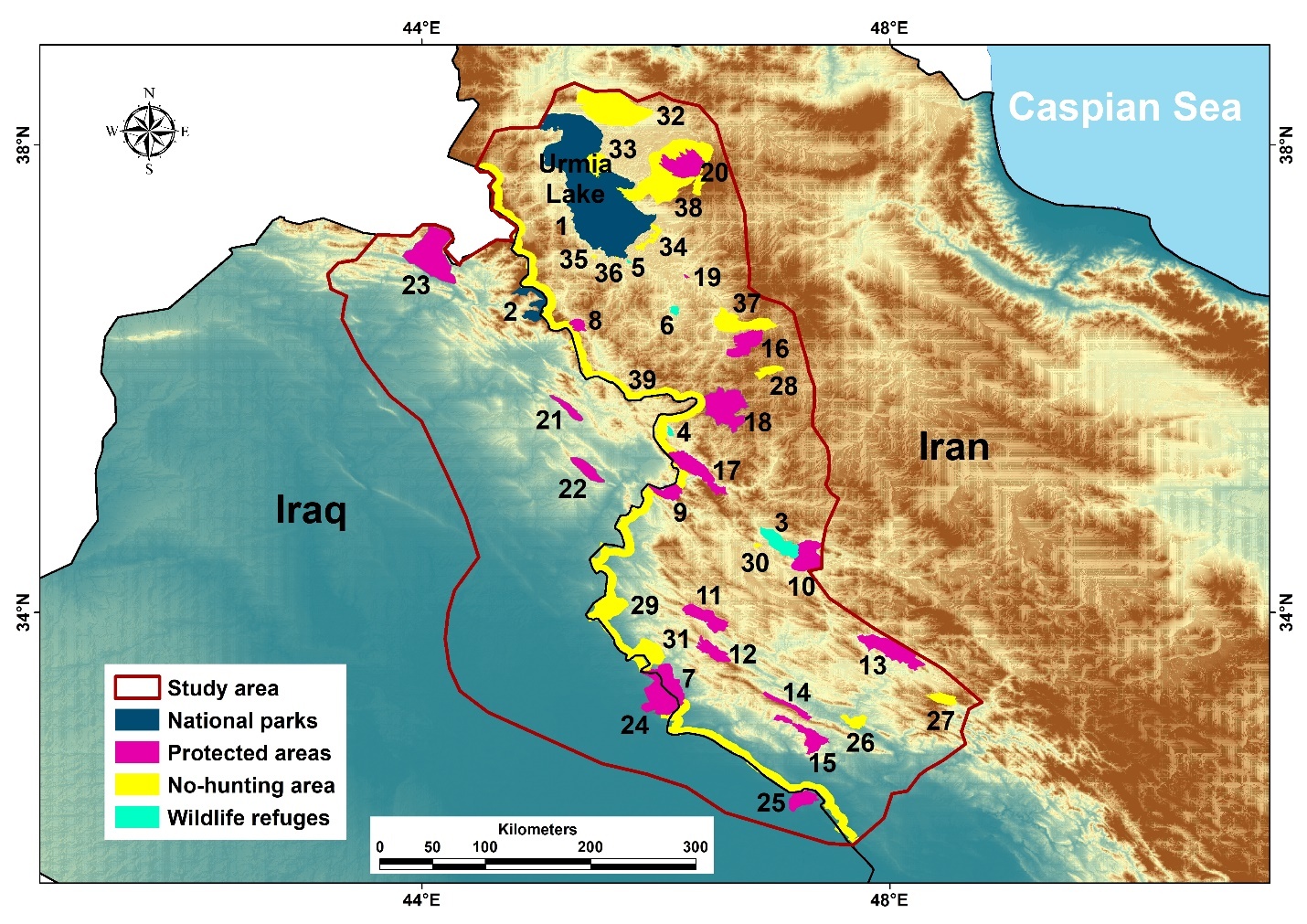


FIGURE S2 Study area along the Iran-Iraq border and conservation areas (conservation area’s name of each number is available in Table S1).


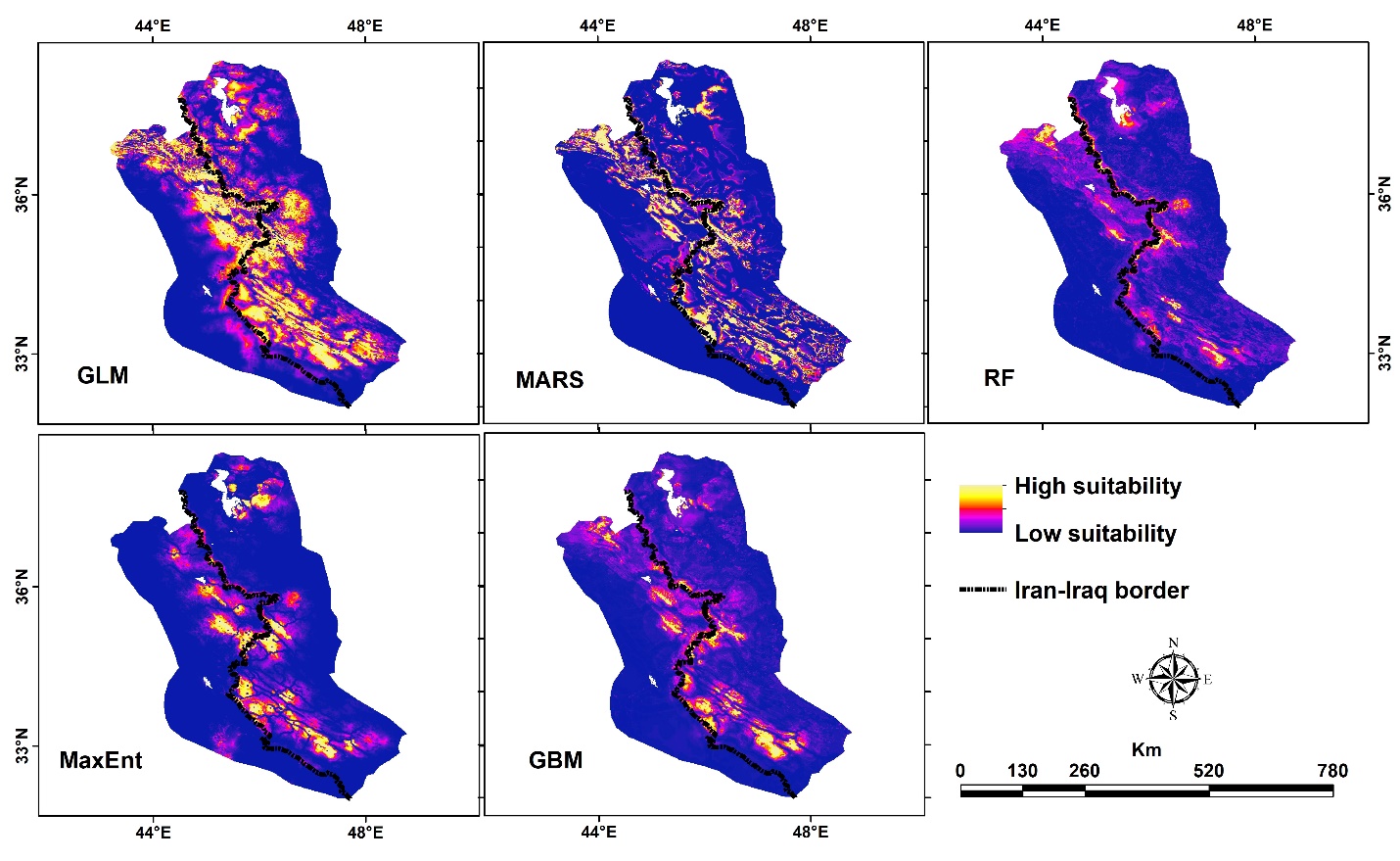


FIGURE S3 Optimal five habitat suitability models for the Persian leopard in the study area.


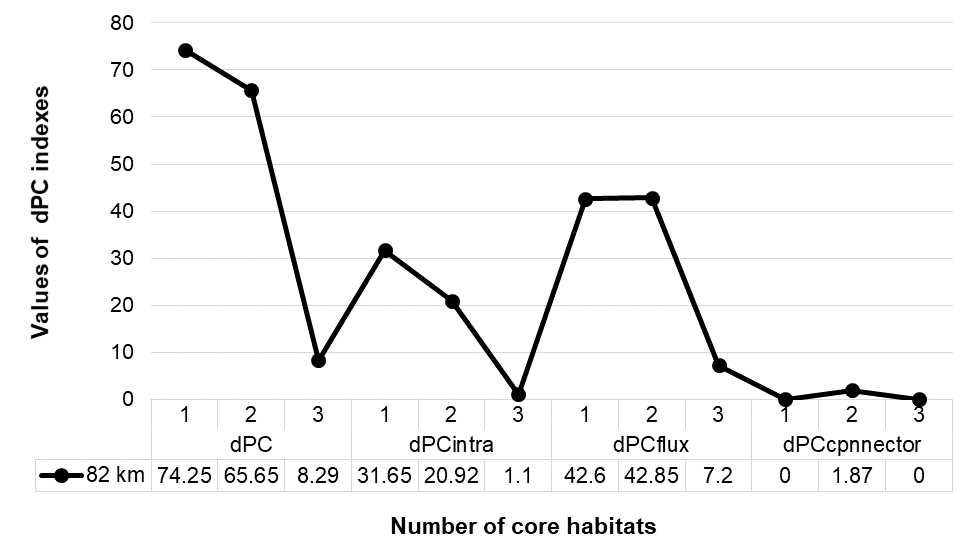


FIGURE S4 Values of dPC index and its three fractions (intra, flux and connector) calculated for predicted three core habitats at the dispersal scenario of 82 km (number of core habitats are available in Figure 4)

**APPENDIX**

**TABLES**

TABLE S1 List of conservation network in the study area including two parts of Iran and Iraq (Numbers in parentheses are the areas based on km^2^ and each CA number in the Figure S2, respectively).

| Conservation network | Iran | Iraq |
| --- | --- | --- |
| National parks | Urmia NP (5410.6, 1) | Sakran Mountains NP (341.51, 2) |
| Wildlife refuges | Bisotoon WR (406.51, 3) | - |
|  | Zarivar WR (32.92, 4) |  |
|  | Kanibarazan WR (48.79, 5) |  |
|  | Dasht-e- Sota and Hamimian WR (8.76, 6) |  |
| Protected areas | Kolak PA (588.47, 7) | Piramagroon PA (167.37, 21) |
|  | MirAbad PA (112.69, 8) | Qara Dagh PA (253.45, 22) |
|  | Buzin and Marakhil Protected Area (235.46, 9) | Barzan area and Gali Balnda PA (1148.22, 23) |
|  | Bisotoon PA (520.97, 10) | Badra and Zurbatiyah PA (669.59, 24) |
|  | Ghalajeh PA (426.05, 11) | Teeb Qasis and Zubaidaat PA (285.77, 25) |
|  | Manesht and Gelarang PA (293.1, 12) |  |
|  | SefidKuh PA (709.52, 13) |  |
|  | KabirKuh PA (184.53, 14) |  |
|  | DinarKuh PA (405.67, 15) |  |
|  | Abdolrezagh PA (439.5, 16) |  |
|  | Shaho-Kosalan PA (572.35, 17) |  |
|  | Chehelcheshmeh and Saral PA (933.76, 18) |  |
|  | Norouzlu PA (8.3, 19) |  |
|  | Sahand PA (660.54, 20) |  |
| No-hunting area | Pole Dokhtar Wetlands NHAs (181.44, 26) | - |
|  | Hashtad Pahlo NHAs (184.73, 27) |  |
|  | Zarin NHAs (148.3, 28) |  |
|  | Zelehzard NHAs (260.09, 29) |  |
|  | Hashilan NHAs (20.31, 30) |  |
|  | Bina and Bijar and Chakoor NHAs (382.39, 31) |  |
|  | Mishodagh NHAs (1293.49, 32) |  |
|  | Eslami Island NHAs (265.77, 33) |  |
|  | Qare Qeshlaq NHAs (138.65, 34) |  |
|  | Hasanlu Wetland NHAs (11.45, 35) |  |
|  | Garde Qite Maymand NHAs (3.74, 36) |  |
|  | Bayan NHAs (588.02, 37) |  |
|  | Sahand NHAs (1559.42, 38) |  |
|  | the width of seven kilometers on the border of the Iranian side (6958.02, 39) |  |

TABLE S2 Sources of occurrence points of the Persian leopard in the study area.

| Sources of occurrence points | Number of occurrence points in the study area | |
| --- | --- | --- |
|  | Iran | Iraq |
| Direct observation | 3 | 1 |
| Camera traps | 3 | 1 |
| Scats | 9* | - |
| Foot signs | 6* | - |
| Dead body (because of diseases, gun shooting or poisoning) | 2 | 6 |

* Confirmed by the Department of Environment of Iran

TABLE S3 Environmental variables used in the previous studies and their effects on the habitat suitability of the Persian leopard in Iran.

| Variables | | Important variables | References |
| --- | --- | --- | --- |
| Category | Name |  |  |
| Topography | roughness | roughness (1), BIO4 (2), NDVI (3), BIO1 (4), Human footprint (5) and mosaic of shrubland–forest (6) | Farhadinia et al., 2015 |
| Climate | annual mean temperature (BIO1), temperature seasonality (BIO4), annual precipitation (BIO12) and Precipitation seasonality (BIO1 |  |  |
| Land-cover | forest cover, mosaic of shrubland–forest, shrubland, mosaic of grassland–shrubland, closed to open herbaceous vegetation, mosaic of grassland–cropland, cropland and NDVI |  |  |
| Anthropogenic  variable | human footprint |  |  |
| Topography | slope, aspect | presence of *Capra aegagrus* (1), slope (2), distance to villages (3) and presence of *Ovis orientalis* (4) | Ashrafzadeh et al., 2018 |
| Climate | temperature seasonality (BIO4), temperature annual range (BIO7), mean temperature of the driest  quarter (BIO9), annual precipitation (BIO12) and precipitation of the driest quarter (BIO17) |  |  |
| Land-cover | land cover, distance to rivers |  |  |
| Anthropogenic variables | human footprint, distance to roads, distance to villages |  |  |
| Prey availability | presence of *Ovis orientalis*, presence of *Capra aegagrus* |  |  |
| Topography | digital elevation model, topographic position index and roughness | prey density (1), roughness (2), shrubland  density (3) and road density (4) | Khosravi et al., 2018 |
| Climate | annual precipitation (BIO12) |  |  |
| Land-cover | poor  range (sparse vegetation with density ≤25%), moderate canopy  rangeland (mixture of grassland–scrubland with density ≥25%), shrubland (patches covered by scrubs–shrubs with canopy cover  ≥10%), bareland (uncovered areas including sand dunes and  salty lands) and NDVI |  |  |
| Anthropogenic variables | human  settlements, villages and roads density |  |  |
| Prey availability | occurrence four ungulate species including wild sheep  (*Ovis orientalis*), goitered gazelle (*Gazella subguturosa*), wild goat (*Capra aegagrus*) and  jebeer gazelle (*Gazella bennetti*) |  |  |
| Topography | roughness | roughness, distance to woodlands, annual mean temperature (BIO1) | Hosseini et al., 2019 |
| Climate | annual mean temperature (BIO1), annual precipitation (BIO12) |  |  |
| Landcover | distance to agriculture, distance to scrubland, distance to woodland, distance to range area, distance to rocky area, NDVI |  |  |
| Anthropogenic  variable | distance to urban area, distance to rural area, distance to highway, distance to road, distance to mine |  |  |
| Topography | roughness | roughness (1), percentage of landscape (sparse rangeland with a density of ≤ 20) (2) and village density (3) | Khosravi et al., 2019 |
| Climate | annual precipitation (BIO12) |  |  |
| Land-cover | percentage of landscape (Bareland), percentage of landscape (sparse rangeland with a density of ≤ 20) and area-weighted mean patch radius of gyration (mixture of grassland–scrubland with a density of ≥25) |  |  |
| Anthropogenic  variable | village density |  |  |
| Topography | digital elevation map, slope, roughness, compound topographic index | Mean annual temperature (BIO1) (1), temperature seasonality (BIO4) (2) and digital elevation map (3) | Ashrafzadeh et al., 2020 |
| Climate | mean annual temperature  (BIO1), annul precipitation (BIO12), temperature seasonality  (BIO4), and precipitation seasonality (BIO15) |  |  |
| Land-cover | forest, shrubland, grassland–scrubland with a density  of ≥25%, sparse rangeland with a density of ≤20%, cropland, urban area, and bareland ( composed of sand dunes and salty lands) |  |  |
| Protection | level of habitat protection |  |  |
| Anthropogenic  variable | human footprint |  |  |
| Topography | digital elevation map, roughness | not detected | Ahmadi et al., 2020 |
| Climate | - |  |  |
| Land-cover | dense to moderate density forest, scrub-shrub land, sparse vegetation, unvegetated bare  lands (e.g. playas and salt lands) and cropland |  |  |
| Anthropogenic  variable | human footprint |  |  |
| Prey availability | occurrence of roe  deer (*Capreolus capreolus*), occurrence of red deer  (*Cervus elaphus*), occurrence of bezoar goat (*Capra*  *aegagrus*), occurrence of wild sheep (*Ovis orientalis*), occurrence of goitered gazelle (*Gazella subgutturosa*) and occurrence of chinkara (*G. bennettii*) |  |  |
| Topography | roughness | prey availability (1), roughness (2), annual mean temperature (BIO1), temperature seasonality (BIO4), and precipitation of the wettest season (BIO16) | Khosravi et al., 2021 |
| Climate | annual mean temperature (BIO1), temperature seasonality (BIO4), min temperature of coldest month (6), mean temperature of warmest season (BIO10), mean temperature of coldest season (BIO11), precipitation seasonality (BIO15), precipitation of wettest season (BIO16), and precipitation of the driest season (BIO17) |  |  |
| Land-cover | - |  |  |
| Anthropogenic  variable | - |  |  |
| Prey availability | prey abundance of Jebeer gazelle (*Gazella bennettii*), goitered gazelle (*Gazella subgutturosa*), wild goat (*Capra*  *aegagrus*), mouflon (*Ovis orientalis*) |  |  |

TABLE S4 Selected variables for habitat modeling of the Persian leopard in the study area with correlation coefficient of < 70% and the VIF of < 3.

| Variables (with correlation coefficient of <70%) | VIF |
| --- | --- |
| Roughness | 1.27 |
| Annual mean temperature (BIO1) | 1.61 |
| Annual precipitation (BIO12) | 1.98 |
| Vegetation/cropland density | 1.73 |
| Grassland/forest-shrubland density | 1.55 |
| NDVI | 1.45 |
| Distance to rivers | 1.28 |
| Distance to conservation areas | 1.31 |
| Distance to roads | 1.25 |
| Distance to villages | 1.59 |

TABLE S5 AUC and TSS of 10 different models in the primary habitat suitability of the Persian leopard in the study area.

|  | GLM* | GAM | MARS* | FDA | RF* | MaxEnt* | GBM* | CTA | ANN | SRE |
| --- | --- | --- | --- | --- | --- | --- | --- | --- | --- | --- |
| AUC | 0.905 | 0.734 | 0.91 | 0.836 | 0.928 | 0.906 | 0.922 | 0.759 | 0.81 | 0.767 |
| TSS | 0.771 | 0.525 | 0.801 | 0.65 | 0.821 | 0.792 | 0.81 | 0.533 | 0.63 | 0.47 |

* Optimal models which have been selected for final habitat modeling.

TABLE S6 Variable contribution (mean and standard deviation of five optimal models) in the habitat modeling of the Persian leopard in the study area.

|  | Roughness | Annual mean temperature (BIO1) | Annual precipitation (BIO12) | Vegetation/cropland density | Grassland/forest-shrubland density | NDVI | Distance to rivers | Distance to conservation areas | Distance to roads | Distance to villages |
| --- | --- | --- | --- | --- | --- | --- | --- | --- | --- | --- |
| Mean | 16.4 | 5.8 | 12.9 | 11.1 | 6.9 | 7.1 | 10.9 | 13.1 | 6.7 | 9.1 |
| SD | 3.9 | 2.2 | 3.3 | 2.3 | 2.4 | 1.4 | 3.4 | 1.9 | 2.9 | 1.7 |

TABLE S7 Core habitats and the number of different CAs inside each identified core for the Persian leopard in the study area.

| Estimated dispersal distance | Core | | Number inside cores | | | | |
| --- | --- | --- | --- | --- | --- | --- | --- |
|  | Number | Area (km^2^) | NP | PA | WR | NHA | |
| 82 km | 1 | 11456.27 | - | 4 | - | | 3* |
|  | 2 | 9316.08 | - | 3 | - | | -* |
|  | 3 | 2137.53 | - | - | - | | - |

* Including the width of seven kilometers on the border of the Iranian side.

TABLE S8 Properties of the predicted core habitats and corridors for the Persian leopard and coverage with the existing CAs in the study area

| Determined dispersal distance (km) | Area of core habitats (km^2^) | Protected core habitats | | Area of Corridors (km^2^) | Protected corridors | |
| --- | --- | --- | --- | --- | --- | --- |
|  |  | Area (km^2^) | % |  | Area (km^2^) | % |
| 82 | 22909.87 | 4461.39 | 19.47 | 7800.27 | 1553.9 | 19.92 |
